# Facet Preferencing by Chemical Substitution Controls Semi-Hydrogenation Selectivity in Ternary Pyrite-Type Intermetallic Compounds

**DOI:** 10.1021/acscatal.5c08855

**Published:** 2026-01-28

**Authors:** Mustafa Eid, Jin Li, Nilanjan Roy, Kathryn MacIntosh, Michael J. Janik, Robert M. Rioux

**Affiliations:** † Department of Chemistry, 8082 Pennsylvania State University, University Park, Pennsylvania 16802, United States; ‡ Department of Chemical Engineering, Pennsylvania State University, University Park, Pennsylvania 16802, United States

**Keywords:** intermetallics, chemical substitution, facet, acetylene, hydrogenation, selectivity

## Abstract

Intermetallic compounds serve as model catalysts for selective hydrogenation reactions, offering precise control over the active site composition(s), geometric and electronic structure. The addition of a third element to form a ternary intermetallic alters the exposed crystal facet(s), demonstrating a strategy to impart improved catalytic behavior in intermetallic catalysts. The site-specific substitution of a small fraction of Pd atoms with Au in pyrite-type PdSb_2_ results in the preferential exposure of the (100) facet over the (111) facet. Electron back scattered diffraction and density functional theory calculations confirm the facet change upon the substitution of Pd with Au to form the ternary Pd_1–*x*_Au_*x*_Sb_2_ (0.075 ≤ *x* ≤ 0.25). The (100) facet demonstrates higher net alkene selectivity due to significantly weaker alkene binding compared to the (111) facet. Distinct from our prior work on chemical substitution to directly alter the active site composition, this work demonstrates the indirect modification of active sites via preferential facet exposure.

## Graphical abstract

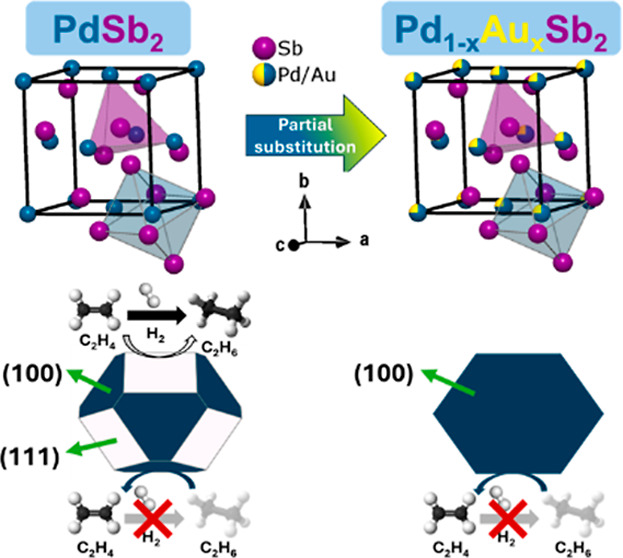

## 1 Introduction

Alloying a catalytically active metal with different elements impacts its catalytic activity and selectivity due to the modification of its geometric and/or electronic states.^1,2^ A common example are solid solutions of Pd_1–*x*_Ag_*x*_, which have been used industrially for acetylene semihydrogenation since they showed higher ethylene selectivity compared to pure Pd.^3,4^ Such improvement in selectivity was attributed largely to an electronic effect via an electron transfer from Ag to Pd upon alloying.^5,6^ However, other studies indicated the isolation of Pd active sites (geometric effect) may promote ethylene desorption, thus improving selectivity.^7^ Similarly, Pd_1–*x*_Au_*x*_ alloys have also been shown to exhibit enhanced selectivity for acetylene hydrogenation compared to monometallic Pd.^8–10^ While Au mainly led to the isolation of Pd atoms which suppressed overhydrogenation, this does not preclude electronic modification of Pd upon alloying with Au.^11,12^ The improvement in catalytic performance over these Pd_1–*x*_Ag_*x*_ and Pd_1–*x*_Au_*x*_ alloys is likely due to a combination of both geometric and electronic effects. The deconvolution of geometric and electronic effects in solid solution alloys, such as Pd_1–*x*_Ag_*x*_, is challenging due to the random distribution of elements, which will likely result in a variety of active site configurations (multinomial distribution) contributing to the overall catalytic behavior.^13,14^

In contrast to solid solution alloys, intermetallic compounds have been studied as model catalysts for acetylene semihydrogenation since they offer the flexibility to modify the active site composition and configuration due to the specific site occupation of metals.^2,15^ The complete or partially ordered atomic arrangement in intermetallics facilitates independent influence of electronic and geometric effects, allowing for the development of rationally designed catalysts for semihydrogenation. Intermetallic catalysts have been widely explored as selective catalysts for acetylene semihydrogenation through active site tuning.^16–24^ An example of the precise control over active site composition and configuration was the use of the Pd–Zn γ-brass for acetylene semihydrogenation.^18^ By tuning the stoichiometry, the exposure of Pd monomer and trimer sites can be controlled. The Pd trimer sites in Pd_9_Zn_43_ demonstrated lower selectivity compared to Pd monomers in Pd_8_Zn_44_ due to at least 10^6^ times faster rate of ethylene hydrogenation on the trimer sites. The inclusion of a coinage metal resulted in the formation of Pd–M–Pd ensembles (M = Cu, Ag, Au) within the majority Zn γ-brass phase, which demonstrated intermediate catalytic activity and selectivity between Pd monomer and Pd trimers. Armbrüster et al. found ethylene selectivity increased over Pd–Ga based intermetallics compared to pure Pd.^22^ The authors explained the increased selectivity was due to a combination of an electronic modification of Pd upon alloying with Ga, and a geometric effect through the isolation of Pd active sites by Ga. Tsai and co-workers demonstrated the substitution of Mn for Fe in Co_2_Mn_*x*_Fe_1–*x*_Ge Heusler alloys led to higher alkene selectivity at complete alkyne conversion compared to the parent Co_2_MnGe and Co_2_FeGe.^24^ The improvement in semihydrogenation selectivity was attributed to changes in the electronic structure (electronic effect) upon the site-specific substitution of Fe with Mn.

Modulating the exposed crystal facet(s) is another approach to impact catalytic activity and selectivity. Somorjai and co-workers studied the catalytic activity of different Pd single-crystal surfaces for acetylene hydrogenation and cyclotrimerization.^25^ Pd(111) was the most catalytically active surface compared to Pd(100) and (110) surfaces. In another study, Maier et al. found the selectivity toward *cis*-hexene in 2-hexyne hydrogenation over Pd single crystals was greatly dictated by the orientation of the crystal facet exposed.^26^ Pd(111) demonstrated high selectivity toward *cis*-hexene (87%) compared to Pd(110), which showed poorer selectivity (37%). Kim and co-workers exploited a colloidal synthesis method to prepare cubic and spherical Pd nanoparticles (NPs), exposing different facets.^27^ Cubic Pd NPs exposed (100) facets and demonstrated higher catalytic activity and better ethylene selectivity compared to the spherical Pd NPs with predominantly (111) facets. Zhang et al. adopted a similar strategy for Pd_3_Pb intermetallic nanocubes to study how facet concavity impacted phenylacetylene semihydrogenation.^28^ Variation in the degree of concavity led to the exposure of three different surfaces, namely (111), (110) and (100), which demonstrated different catalytic activity and selectivity. Among the three surfaces, (111) had the highest catalytic activity for phenylacetylene semihydrogenation but the lowest styrene selectivity. On the contrary, (100) demonstrated the highest selectivity but the lowest catalytic activity, while (110) showed high catalytic activity without sacrificing the high selectivity.

Although colloidal synthesis of intermetallic nanoparticles can allow for some control over the exposed facets, it requires the use of organic ligands (capping agents) to stabilize the nanoparticles, which could potentially alter the catalytic activity and selectivity of the active sites. Moreover, the removal of capping agents from the surface of intermetallic nanoparticles typically requires severe conditions, which may result in oxidation or surface segregation of the intermetallic compound and/or alteration of the morphology of the particles.^29^ Consequently, it may be challenging to distinguish whether the observed catalytic behavior is due to the specific surface facet exposed on the intermetallic particles or the presence of capping agent on the surface. To the best of our knowledge, there has been no report on the preferential exposure of facets in intermetallics dictated by a simple metal substitution, without the need for colloidal syntheses or epitaxial growth of thin films.^26,30^

We chose pyrite-type PdSb_2_ (Figure 1a) as a parent intermetallic system to study the geometric and electronic effects of alloying on Pd active sites. Pd is octahedrally coordinated to six Sb atoms in this structure, leading to surfaces where all Pd atoms are isolated as monomers. Since Au displays the same site occupation as Pd, the partial substitution of Pd with Au can lead to the formation of a stable ternary pyrite-type structure. The substitution of Pd with Au does not impact the composition or configuration of surface Pd active sites due to their isolation as monomers but instead alters the predominant exposed facet. Competitive acetylene-propylene hydrogenation was employed as a probe reaction to mimic the industrial operating conditions for the removal of acetylene impurities from crude ethylene, where selective hydrogenation to ethylene without overhydrogenation to ethane is crucial. Preferential exposure of the specific (100) facet resulted in an enhancement of the ethylene selectivity during acetylene semihydrogenation, as confirmed by the agreement between characterization, density functional calculations (DFT), and kinetic studies.

**Figure 1:**
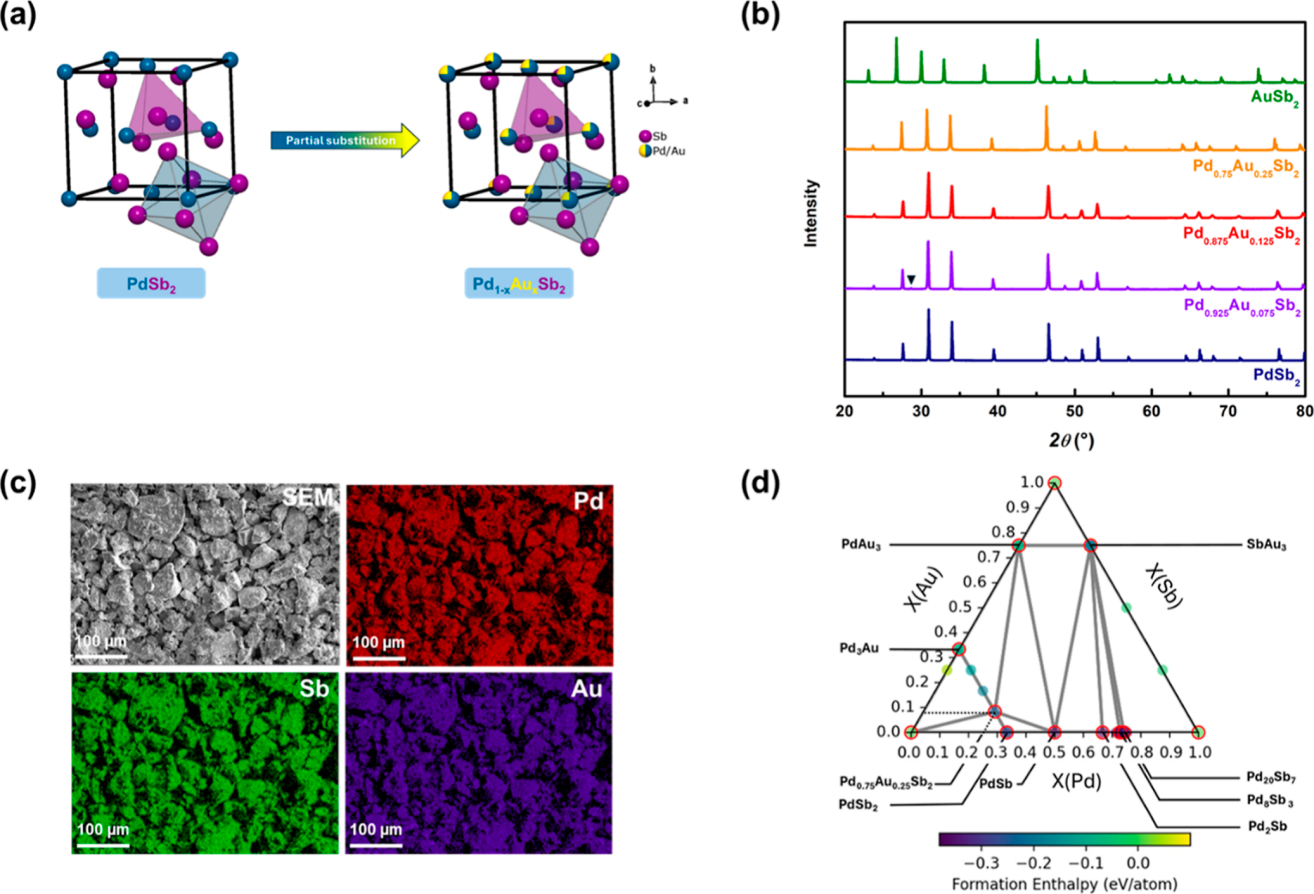
(a) The unit cell of pyrite-type PdSb_2_ and Pd_1–*x*_Au_X_Sb_2_. (b) Powder XRD of PdSb_2_, Pd_0.925_Au_0.075_Sb_2_, Pd_0.875_Au_0.125_Sb_2_, Pd_0.75_Au_0.25_Sb_2_ and AuSb_2_. The upside-down triangle represents an impurity peak of Sb. (c) EDS-SEM map of Pd_0.75_Au_0.25_Sb_2_ demonstrating the homogeneous atomic distribution of Sb (green color), Pd (red color), and Au (deep violet). (d) Density functional theory based ternary phase diagram of the Pd–Au–Sb intermetallic system with calculated formation enthalpies at 0 K. Compositions that lie on the convex hull (thermodynamically stable phases) are highlighted with markers outlined by a red contrasting border. The color scale indicates the formation enthalpy in eV/atom, with more negative values (violet) corresponding to greater thermodynamic stability. Compositions with formation enthalpies above the convex hull (yellow and green) correspond to metastable or unstable phases. The dashed line denotes the pseudobinary compositional path Pd_1–*x*_Au_*x*_Sb_2_, illustrating Pd substitution by Au from the PdSb_2_ binary compound.

## 2 Experimental and Computational Methods

### 2.1 Synthesis of Pyrite-Type PdSb_2_, Pd_1–*x*_Au_*x*_Sb_2_ and AuSb_2_

Intermetallic PdSb_2_ and Pd_1–*x*_Au_*x*_Sb_2_ were synthesized using a high temperature solid-state approach. A targeted amount of Pd powder (Strem Chemicals, 99.999%), Au powder (Sigma-Aldrich, ≥99.9%) and Sb powder (Thermo Scientific, 99.999%) were placed into a homemade quartz ampoule, evacuated to a pressure of ∼ 2 × 10^–3^ mbar and flame-sealed under vacuum. The ampoule was heated in a muffle furnace (Thermo-Scientific, Lindberg Blue M) from room temperature to 1100 °C at a ramp rate of ∼ 1 °C/min, held at 1100 °C for 24 h, cooled to 600 °C at a ramp rate of ∼ 0.33 °C/min and finally held at 600 °C for 100 h. The resultant ingot was then crushed into a powder using a mortar and pestle. After grinding the ingot into a powder, the samples were reannealed under vacuum at 500 °C for 120 h.

The AuSb_2_ intermetallic compound was synthesized using the same approach described above, but with a slightly different temperature program. A targeted amount of Au powder (Sigma-Aldrich, ≥99.9%) and Sb powder (Thermo Scientific, 99.999%) were placed into a homemade quartz ampoule, evacuated to a pressure of ∼ 2 × 10^–3^ mbar and flame-sealed under vacuum. The ampoule was heated in a box furnace from room temperature to 665 °C at a ramp rate of ∼ 1 °C/min, held at 665 °C for 24 h, raised to 1072 °C at a ramp rate of ∼ 1 °C/min, held at 1072 °C for 10 h, cooled to 350 °C with a ramp rate of ∼ 0.33 °C/min and finally held at 350 °C for 100 h. The resultant ingot was crushed into a powder using a mortar and pestle. After grinding to a powder, the sample was again annealed under vacuum at 350 °C for 100 h.

### 2.2 Characterization of Pyrite-Type PdSb_2_, AuSb_2_ and Pd_1–*x*_Au_*x*_Sb_2_

#### 2.2.1 Powder X-ray Diffraction

X-ray diffraction (XRD) measurements were taken on a Malvern PANalytical Empyrean 3, operating in the θ–2θ Bragg configuration using Cu Kα (α = 1.5406 Å) at 40 kV radiation. Scans were made from 20–80° with a step size of 0.02° at a scan rate of 0.067°/s. Peak fitting and data analysis were performed using the JANA 2006 software.^38^

#### 2.2.2 Single Crystal X-ray Diffraction

The single crystal diffraction data were collected on a Rigaku Micromax 007 rotating anode (copper) generator equipped with an Osmic Varimax VHF monochromator, a universal four-circle Kappa goniometer, and a HyPix-Arc150 area detector. The samples were prepared by breaking the intermetallic ingots into smaller single crystals, which were then mounted on the goniometer to collect the diffraction data. Data collection and subsequent reduction was performed using CrysAlisPro (Rigaku) software. The collected data was further analyzed using the JANA 2006 software to identify structural parameters such as lattice constants, site-occupancy factors (SOFs) and atomic coordinates.

#### 2.2.3 Scanning Electron Microscopy and Energy Dispersive X-ray Spectroscopy

EDS maps were collected using a Verios G4 SEM (Thermo-Scientific) equipped with an Oxford Instrument X-Act EDS detector. Samples were sprinkled onto black carbon tape prior to imaging and then mounted on the sample holder. The EDS maps were generated using the Aztec software package.

#### 2.2.4 Electron Back Scattered Diffraction

EBSD orientation maps were collected in an Apreo S SEM (Thermo-scientific). EBSD (Oxford Nordlys Max2 instrument) was employed to map various domain orientations within the crystals. The sample was tilted to 70° with respect to the horizontal. The diffraction pattern was imaged on a phosphor screen and the image was captured using a low-light CMOS camera. We analyzed 3–5 distinct crystals for each sample to ensure reproducibility.

#### 2.2.5 Surface Area Measurement (Physisorption)

BET (Brunauer–Emmett–Teller) analysis was used to determine the surface area by Kr adsorption at −196 °C on a Micromeritics 3FLEX volumetric adsorption apparatus with a P/P_0_ range between 0.07 and 0.41. Approximately 100–200 mg of each sample was loaded into the sample tube and degassed under vacuum at 240 °C overnight prior to the measurement. The pyrite-type intermetallics had a BET surface area of ∼ 0.2–0.3 m^2^/g, regardless of composition.

#### 2.2.6 Inductively Coupled Plasma-Optical Emission Spectroscopy

The metallic compositions of intermetallic compounds were confirmed by inductively coupled plasma-optical emission spectroscopy (ICP-OES) (Thermo Scientific iCAP 7400). Approximately 10 mg of each intermetallic compound was digested in a 50 mL scintillation vial using concentrated acids (1 mL of HNO_3_, 2.9 mL of saturated HCl and 100 μL of HF). The scintillation vial was capped, sonicated for 30 min, and diluted with 16 mL of DI water. A calibration curve was constructed from the standards and used to determine the quantity of metals in each sample. Standards were made from 1000 μg/mL stock solution of palladium (Inorganic Ventures, 5% HNO_3_), antimony (High-Purity Standards, 5% HNO_3_/0.1% HF), and gold (High-purity Standards, 2% HNO_3_).

### 2.3 Catalytic Kinetics Measurements

Competitive acetylene-propylene hydrogenation was performed in a homemade down-flow reactor. The catalysts were loaded into a quartz reactor tube (O.D. = 0.5″). The reactor tube was placed inside a clam-shell furnace (Applied Test Systems) with a K-type thermocouple in contact with the bottom of the catalyst bed. Catalyst beds were prepared by diluting the active catalyst material in SiO_2_ (Sigma-Aldrich, Davisil grade 62). PdSb_2_ (57–140 mg) was diluted in SiO_2_ to give a volume ratio of ∼ 9–20% PdSb_2_ and 91–80% SiO_2_; Pd_0.925_Au_0.075_Sb_2_ (53–220 mg) was diluted in SiO_2_ to give a volume ratio of ∼ 9–28% Pd_0.925_Au_0.075_Sb_2_ and 91–72% SiO_2_; Pd_0.875_Au_0.125_Sb_2_ (50–250 mg) was diluted in SiO_2_ to give a volume ratio of ∼ 9–31% Pd_0.875_Au_0.125_Sb_2_ and 91–69% SiO_2;_ Pd_0.75_Au_0.25_Sb_2_ (70–320 mg) was diluted in SiO_2_ to give a volume ratio of ∼ 11–35% Pd_0.75_Au_0.25_Sb_2_ and 89–65% SiO_2_; AuSb_2_ (80–200 mg) was diluted in SiO_2_ to give a volume ratio of ∼ 12–25% AuSb_2_ and 88–75% SiO_2_; 0.0175–0.05 mg Pd powder (Strem Chemicals, 99.999%) was diluted in SiO_2_ (volume ratio ∼ 100% SiO_2_). Each catalyst bed was reduced for 8 h at 250 °C in H_2_. H_2_ was sourced from a H_2_ generator (Fuel Cell Store) after which the H_2_ was passed through drierite traps for the removal of moisture and then a liquid nitrogen trap for the removal of H_2_O and O_2_. The estimated purity of H_2_ is 99.9999%. After reduction, the catalyst bed was cooled down to the reaction temperature of 160 °C. The conversion-selectivity behavior over each catalyst bed was examined by varying the total volumetric flow rate between 32, 40, 60, 80, and 100 mL/min with 0.16 kPa acetylene (Praxair, 1% acetylene in helium), 4.8 kPa propylene (Linde, 10% propylene in nitrogen), 32 kPa hydrogen and balance nitrogen. The weight hour space velocity (WHSV) was varied between 0.00033 and 15.51 mmol_acetylene_ g_cat_^–1^ h^–1^. The intrinsic and net ethylene selectivity were calculated as

1 $$intrinsic\;ethylene\;selectivity=\frac{C_{2}H_{4}}{{\left(C_{2}H_{2}\right)}_{in}-{\left(C_{2}H_{2}\right)}_{out}}$$

2 $$net\;ethylene\;selectivity=\frac{C_{2}H_{4}-C_{3}H_{8}}{{\left(C_{2}H_{2}\right)}_{in}-{\left(C_{2}H_{2}\right)}_{out}}$$

Calculation of the intrinsic selectivity only considers the selectivity to ethylene over ethane or C_4_ products formed from acetylene, while the net selectivity accounts for the hydrogenation of alkene added to the feed, using the surrogate, propylene, to represent ethylene cofed with the acetylene. The use of propylene allowed for the determination of the net ethylene selectivity via gas chromatography, in contrast to our prior work that utilized isotopically labeled ^13^C_2_H_4_ to differentiate intrinsic and net selectivity values.^18^ We report the values of intrinsic and net selectivity once the catalyst bed reached steady state (Figure S1).

Acetylene hydrogenation was performed in the same reactor system. Catalyst beds were intermetallic samples diluted with SiO_2_. PdSb_2_ (61 mg) was diluted in SiO_2_ (volume ratio of ∼ 10% PdSb_2_: 90% SiO_2_); Pd_0.925_Au_0.075_Sb_2_ (46.5 mg) was diluted in SiO_2_ to (volume ratio of ∼ 8% Pd_0.925_Au_0.075_Sb_2_: 92% SiO_2_); Pd_0.875_Au_0.125_Sb_2_ (45 mg) was diluted in SiO_2_ to (volume ratio of ∼ 8% Pd_0.875_Au_0.125_Sb_2_: 92% SiO_2_); Pd_0.75_Au_0.25_Sb_2_ (62 mg) was diluted in SiO_2_ to (volume ratio of ∼ 10% Pd_0.75_Au_0.25_Sb_2_: 90% SiO_2_). The apparent activation barrier for acetylene hydrogenation over each catalyst was measured at a temperature range of 145–170 °C after reducing the catalyst at 250 °C for 8 h. The total volumetric flow rate was 60 mL/min with 0.325 kPa acetylene (Praxair, 1% acetylene in helium), 32.5 kPa hydrogen and balance nitrogen. Acetylene conversion was limited to differential conversion (<10%) for all temperatures.

Measurements of alkene (ethylene/propylene) reaction orders were conducted in the same reactor setup. PdSb_2_ (300 mg) was diluted in SiO_2_ (volume ratio of ∼ 34% PdSb_2_: 66% SiO_2_); Pd_0.925_Au_0.075_Sb_2_ (220 mg) was diluted in SiO_2_ to (volume ratio of ∼ 28% Pd_0.925_Au_0.075_Sb_2_: 72% SiO_2_); Pd_0.875_Au_0.125_Sb_2_ (250 mg) was diluted in SiO_2_ to (volume ratio of ∼ 31% Pd_0.875_Au_0.125_Sb_2_: 69% SiO_2_); Pd_0.75_Au_0.25_Sb_2_ (300 mg) was diluted in SiO_2_ to (volume ratio of ∼ 34% Pd_0.75_Au_0.25_Sb_2_: 66% SiO_2_). The reaction orders were measured over each catalyst at 160 °C after reducing the catalyst at 250 °C for 8 h. The alkene orders were measured by varying the pressure of propylene (Linde, 10% propylene in nitrogen) or ethylene (Linde, 10% ethylene in nitrogen) between 1.58 and 7.88 kPa while the H_2_ pressure was held at 31.5 kPa. A Shimadzu Nexis 2030 Series gas chromatograph (GC) with a Restek Alumina BOND/Na_2_SO_4_ column was used to quantify the gases present in the product stream.

### 2.4 Computational Methods

#### 2.4.1 Electronic Structure Calculations

All electronic structure calculations were performed using the Vienna ab initio simulation package (VASP).^31–33^ The interactions between core and valence electrons were treated using the projector augmented-wave (PAW) method.^34^ A plane-wave basis set with a kinetic energy cutoff of 450 eV was employed for the valence electrons. The exchange–correlation functional was modeled by the Perdew–Burke–Ernzerhof (PBE) formulation within the generalized gradient approximation (GGA).^35^

For both bulk and surface calculations, the *k*-point grids were generated based on the reciprocal lattice dimensions. For bulk structures, the *k*-point grid densities along the a, b, and c lattice vectors were set to the nearest integers of 40/a, 40/b, and 40/c, respectively. Both the lattice parameters and atomic positions were fully optimized, with the atom force criteria below 0.05 eV/Å. For surface calculations, the *k*-point grid densities along the in-plane directions were similarly set to the nearest integers of 40/a and 40/b, where a and b are the respective lattice constants in Å. Structural optimizations were carried out until the forces on all unconstrained atoms were below 0.05 eV/Å. Transition states were identified using the climbing image nudged elastic band (CI-NEB) method, with the criterion for a transition state being a maximum force along the reaction coordinate below 0.05 eV/Å.^36^

#### 2.4.2 Formation Energies

The computational formation enthalpy of a compound was defined as the energy difference relative to its constituent elements in their most stable bulk phases. For compounds composed of Pd, Au, and Sb, the formation enthalpy per atom is calculated as

3 $$\Delta H^{formation}=\frac{E_{Pd_{x}Au_{y}Sb_{z}}^{Bulk}-xE_{Pd}^{bulk}-yE_{Au}^{bulk}-zE_{Sb}^{bulk}}{x+y+z}$$

where $E_{Pd_{x}Au_{y}Sb_{z}}^{Bulk}$ is the total energy of the compound calculated by DFT, *E*_Pd_^bulk^, *E*_Au_^bulk^, *E*_Sb_^bulk^ are the DFT energies of pure Pd, Au, and Sb in their most stable bulk structures, and *x*, *y*, and *z* represent the respective atomic counts of Pd, Au, and Sb in the compound.

For the ternary Pd–Au–Sb phase diagram construction, the binary formation energies of Pd–Au, Pd–Sb, and Au–Sb systems were obtained from the Materials Project database to ensure consistency and comparability across all systems.^37^

#### 2.4.3 Surface Energies

Surface energies were computed to identify the most stable facets of the Pd_1–*x*_Au_*x*_Sb_2_ intermetallic compound, focusing on low-index surfaces with Miller indices (h, k, l) ≤ 2. The slab models used for surface energy calculations were constructed symmetrically, exposing identical surfaces on both the top and bottom faces. Since the surface stoichiometries of most slabs deviated from the bulk Pd_1–*x*_Au_*x*_Sb_2_ composition, a reference energy for the “excess” elements was required. In the case of Pd_0.75_Au_0.25_Sb_2_, the bulk energies of fcc Pd and fcc Au were used as reference states for the excess atoms. The surface energy expressions are given by

4 $$E^{surface}=\frac{E^{{{Pd}_{a}{Au}_{b}{Sb}_{c}}^{surface}}-\frac{c}{z}E^{{{Pd}_{x}{Au}_{y}{Sb}_{z}}^{bulk}}-\left(a-\frac{cx}{z}\right)E^{{Pd}^{bulk}}-\left(b-\frac{cy}{z}\right)\left(E^{{Au}^{bulk}}\right)}{2A}$$

where $E^{{{Pd}_{a}{Au}_{b}{Sb}_{c}}^{surface}}$ is the energy of the surface, $E^{{{Pd}_{x}{Au}_{y}{Sb}_{z}}^{bulk}}$ is the energy of the intermetallic bulk, $E^{{Pd}^{bulk}}$ and $E^{{Au}^{bulk}}$ are the energy of pure metal bulk, and A represents the surface area of one side of the symmetric slab.

#### 2.4.4 Adsorption Energies, reaction Energies and Activation Barriers for Acetylene Semihydrogenation

The adsorption energy for surface species is calculated as

5 $$\Delta E_{ads}=E_{slab+i}-E_{slab}-E_{i}$$

where *E*_slab+i_, *E*_slab_, and *E*_i_ are the electronic energies of the metal–adsorbate system, the metal slab, and the adsorbed species in the gas phase, respectively.

Reaction energies (Δ*E*_rxn_) and activation barriers (Δ*E*_act_) are defined as

6 $$\Delta E_{rxn}=E_{final}-E_{initial}$$

where *E*_final_ represents the final state energy and *E*_initial_ is the initial state energy, and

7 $$\Delta E_{act}=E_{TS}-E_{initial}$$

where *E*_TS_ is the energy of the transition state.

## 3 Results and Discussion

### 3.1 Bulk Characterization of Pyrite-type Pd_1–*x*_Au_*x*_Sb_2_ Intermetallics

A high temperature solid-state synthesis was employed to prepare pyrite-type structure PdSb_2_, AuSb_2_ and Pd_1–*x*_Au_*x*_Sb_2_ intermetallics, since it provides precise control over stoichiometry. The substitution of Pd with Au in Pd_1–*x*_Au_*x*_Sb_2_ was varied (where *x* = 0, 0.075, 0.125, 0.25, 0.5, 0.75, and 1). However, only Pd_1–*x*_Au_*x*_Sb_2_ (*x* ≤ 0.25) as well as the parent binary PdSb_2_ and AuSb_2_ intermetallic compounds were successfully synthesized as pure phases. When the Au content in Pd_1–*x*_Au_*x*_Sb_2_ exceeded *x* > 0.25, phase segregation occurred resulting in the presence of a mixture of the binary AuSb_2_ and ternary Pd_0.75_Au_0.25_Sb_2_ phases (Figure S2). Powder X-ray diffraction (XRD) confirmed the crystal structure and phase purity of pyrite-type PdSb_2_, AuSb_2_ and Pd_1–*x*_Au_*x*_Sb_2_ (*x* = 0.075, 0.125 and 0.25) intermetallics (Figure 1b). The ternary Pd_1–*x*_Au_*x*_Sb_2_ compounds have the pyrite-type structure with the cubic space group (*Pa*3̅) like PdSb_2_ and AuSb_2_. Unit cell parameters of pyrite-type Pd_1–*x*_Au_*x*_Sb_2_ determined by single crystal XRD were used to calculate site-occupancy factors (SOFs) within the unit cell (Table S1). Pd and Au were found to occupy the same crystallographic site, resulting in a site-specific replacement of Pd with Au in the ternary Pd_1–*x*_Au_*x*_Sb_2_ intermetallics. The addition of Au in Pd_1–*x*_Au_*x*_Sb_2_ resulted in an increase in the lattice constant from 6.4644 Å to 6.4663 Å, 6.4668 Å, and 6.4912 Å for Pd_0.925_Au_0.075_Sb_2_, Pd_0.875_Au_0.125_Sb_2_, and Pd_0.75_Au_0.25_Sb_2_, respectively, owing to the larger size of the Au compared to Pd. The SOFs for Pd/Au were 0.927/0.073, 0.88/0.12, and 0.77/0.23 are consistent with the nominal values in Pd_0.925_Au_0.075_Sb_2_, Pd_0.875_Au_0.125_Sb_2_, and Pd_0.75_Au_0.25_Sb_2,_ respectively.

Energy dispersive spectrometry-scanning electron microscope (EDS-SEM) showed Pd, Au, and Sb elements homogeneously distributed across the binary PdSb_2_ and AuSb_2_ as well as the ternary Pd_1–*x*_Au_*x*_Sb_2_ (*x* ≤ 0.25) pyrite-type intermetallics (Figures 1c and S3a–e). The atomic compositions of PdSb_2_, AuSb_2_, and Pd_1–*x*_Au_*x*_Sb_2_ obtained from EDS-SEM and ICP-OES matched well with the refined metal occupancies identified by single crystal XRD (Table S2). The data from EDS-SEM maps combined with XRD analysis, which showed minimal impurity phases, demonstrated the prepared samples are homogeneous with high phase purity.

The relative formation energies of binary and ternary pyrite-type PdSb_2_ intermetallics were quantified using DFT calculations (Figure 1d). Binary structures were obtained from the Materials Project database whereas the ternary structures (ranging from PdSb_2_ to AuSb_2_) were generated by enumerating all possible Au substitutions in the pyrite-type PdSb_2_. Among the enumerated ternary structures, the lowest-energy configurations were selected to construct the convex hull, which is used to determine the thermodynamic stability of different compositions. The substitution of Pd with Au in Pd_1–*x*_Au_*x*_Sb_2_ is only favorable up to *x* = 0.25, forming a stable ternary Pd_0.75_Au_0.25_Sb_2_ phase. Beyond *x* = 0.25 in Pd_1–*x*_Au_*x*_Sb_2_, phase segregation is more favorable than further substitution of Au into Pd lattice positions, leading to the formation of separate AuSb_2_ and Pd_0.75_Au_0.25_Sb_2_ phases. The DFT ternary pyrite phase stability range, and preferential substitution of Au at Pd lattice sites, is consistent with experimental findings.

### 3.2 Surface Characterization of Pyrite-type Pd_1–*x*_Au_*x*_Sb_2_ Intermetallics

Surface energy DFT calculations were performed to identify the lowest-energy facet and its most stable termination. All low-index surfaces with Miller indices less than or equal to 2 were enumerated for both PdSb_2_ and Pd_0.75_Au_0.25_Sb_2_ and corresponding Wulff constructions were generated. DFT calculated surface energies for the binary PdSb_2_ compound demonstrated the (111) and (100) surfaces have similar surface energy and are exposed as the lowest energy surfaces, with Pd present as isolated monomers on both surfaces (Figure 2a). The respective surface energies are 0.48 J/m^2^ for the (001) facet, occupying 41.3% of the crystal surface, while the (111) facet has a surface energy of 0.50 J/m^2^, occupying 51.3% of the crystal surface. The inclusion of Au in Pd_1–*x*_Au_*x*_Sb_2_ decreased the surface energy of the (100) facet to 0.27 J/m^2^ with Au inclusion, leading to exposure of only the most stable (100) surface in the Wulff construction and no exposure of the (111) facet (Figure 2b). The lowering of the (100) surface energy does not arise from preferred exposure of Au atoms at the surface, but from a complex cumulative effect that cannot be easily allocated to the specific atom arrangements of the surface. Further analysis indicates the preferential exposure of the (100) facet upon Au substitution arises from a selective stabilization of this facet rather than a destabilization of the (111) facet. While Au substitution induces only a minor change in the surface energy of the (111) facet, it significantly lowers the surface energy of the more open (100) facet. This behavior is consistent with facet-dependent electronic relaxation, as reflected by a much larger work-function increase on the (100) surface (ΔΦ ≈ + 0.25 eV) compared to the (111) surface (ΔΦ ≈ + 0.06 eV) upon Au substitution, indicating a stronger modification of the surface dipole and electrostatic potential on the (100) facet.

**Figure 2:**
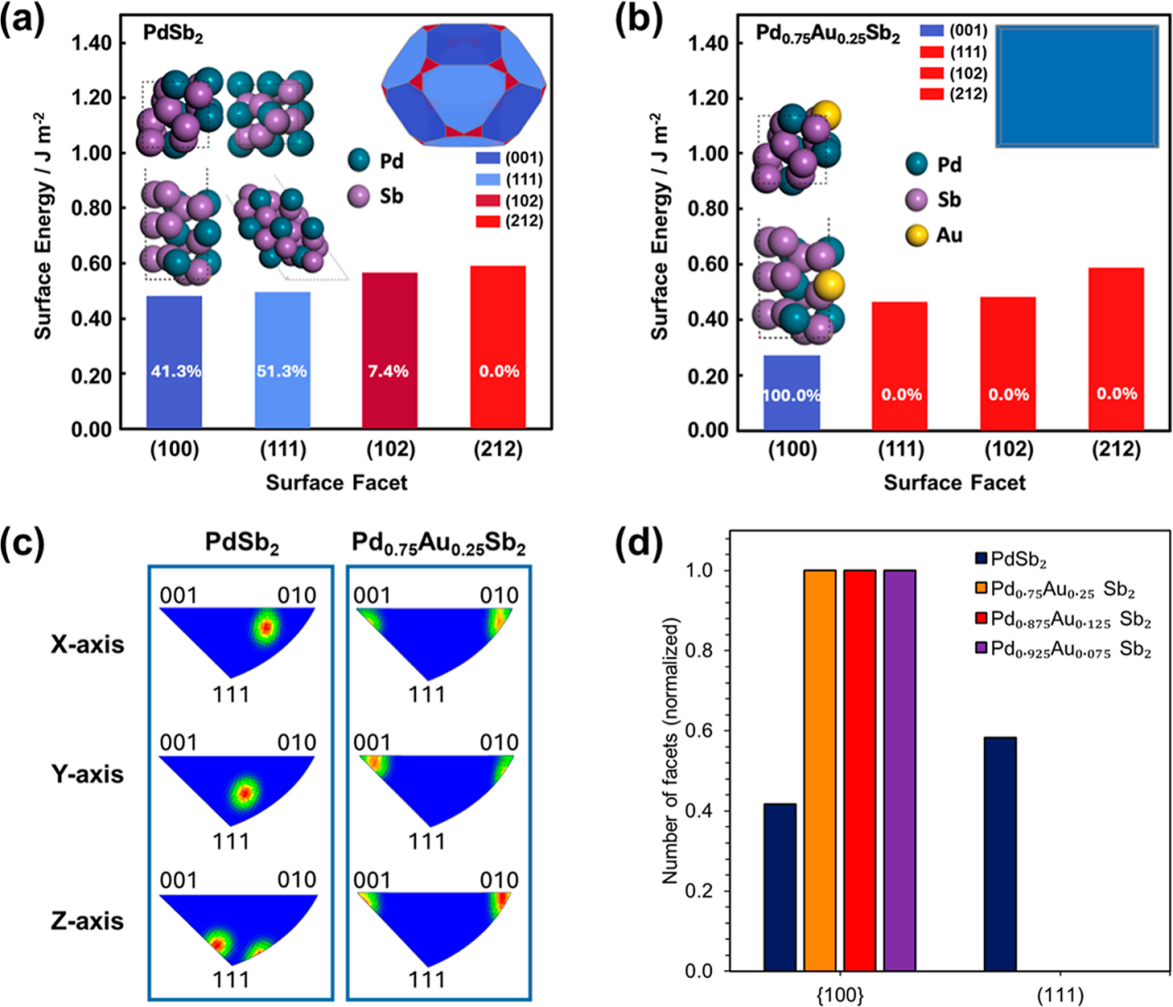
DFT surface energies of low index facets for (a) PdSb_2_ and (b) Pd_0.75_Au_0.25_Sb_2_ along with the most favored termination for each orientation and the corresponding Wulff construction (inset). Numbers in the bar diagram represent the percentage exposure of respective terminations in the Wulff construction. Pd atoms are blue, Sb atoms are violet, and Au atoms are yellow. (c) Inverse pole figure (IPF) 3D crystal orientation maps for PdSb_2_ and Pd_0.75_Au_0.25_Sb_2_. (d) Summary of the crystal facets exposed in Pd_1–*x*_Au_*x*_Sb_2_ samples, as determined experimentally by EBSD analysis, for PdSb_2_ (navy color), Pd_0.925_Au_0.075_Sb_2_ (red color), Pd_0.875_Au_0.125_Sb_2_ (violet color), and Pd_0.75_Au_0.25_Sb_2_ (orange color).

Electron back scattered diffraction (EBSD) was used to identify the crystal facets for comparison with the DFT predictions. Figure 2c and Table S3 display the 3D inverse pole figure (IPF) maps for PdSb_2_ and Pd_1–*x*_Au_*x*_Sb_2_ (*x* = 0.075, 0.125 and 0.25). The (111) and (100) crystal facets were consistently exposed within the crystals of PdSb_2_. On the contrary, the crystals of Pd_1–*x*_Au_*x*_Sb_2_ exposed only the (100) facet (Figure 2d).

Having established the surface structure and facet preferencing, active site compositions were investigated with additional DFT calculations to examine the exchange of subsurface Au atoms with surface Pd monomers. Analysis of the Au distribution revealed Au exists on either the surface or subsurface, since the energy difference between surface terminations with and without Au in the surface layer are negligible.

Both DFT surface energies and EBSD confirm Au substitution of Pd causes preferential faceting leading to exposure of the (100) facet. EBSD indicates even the minimum Au content (*x* = 0.075) explored here causes preferential (100) exposure. However, DFT calculations suggest no strong preference between Au residing on the surface or in subsurface layers. Though Au atoms could be exposed in the (100) ternary surface, isolated Pd sites are expected to serve as the active site regardless of the extent of Au substitution. Subsequent sections examine the impact of Au substitution on hydrogenation catalysis. Importantly, Au substitution creates a large clean-surface stabilization of the (100) facet relative to (111) in Pd_1–*x*_Au_*x*_Sb_2_ (≈0.19 J/m^2^), which is substantially larger than any plausible adsorption-induced correction. Therefore, adsorption under reaction conditions is not expected to modify the relative facet preference and hence does not affect the interpretation of the facet-controlled catalytic behavior discussed below. Furthermore, the catalytic reactivity, shown in Figure S1, remains stable over extended time-on-stream without any noticeable changes in intrinsic and net selectivity. Such behavior is inconsistent with significant restructuring and facet change during reaction.

### 3.3 Kinetics Measurements over Pyrite-Type Pd_1–*x*_Au_*x*_Sb_2_ Intermetallics

Competitive acetylene-propylene hydrogenation was used as a probe reaction to examine the difference in semihydrogenation selectivity (Figure 3a). Propylene acts as a surrogate for ethylene to allow for convenient quantification of intrinsic and net selectivity, without the need for isotopically labeled C_2_ hydrocarbons. Both PdSb_2_ and Pd_1–*x*_Au_*x*_Sb_2_ demonstrated superior intrinsic and net ethylene selectivity compared to the reference material, Pd powder (Figure 3b,c). The intrinsic ethylene selectivity of PdSb_2_ and Pd_1–*x*_Au_*x*_Sb_2_ were comparable. However, Pd_1–*x*_Au_*x*_Sb_2_ demonstrated higher net ethylene selectivity compared to PdSb_2_ at both low and high acetylene conversion. Such improvement in the net selectivity for Pd_1–*x*_Au_*x*_Sb_2_ was insensitive to the Au content between the low and high Au substitution (*x* = 0.075 and 0.25, respectively). Since Pd monomers are the active sites in these intermetallic surfaces, the improvement in the selectivity does not simply correlate with the number of Pd atoms replaced. Instead, a minor substitution of Pd with Au led to selectivity enhancement, which was not further improved upon increased Pd substitution. The intrinsic and net selectivity for AuSb_2_ was not determined since it showed no measurable acetylene conversion at the temperatures examined. The inactivity of AuSb_2_ suggests any exposed Au atoms are unlikely to participate in the active site for hydrogenation.

**Figure 3:**
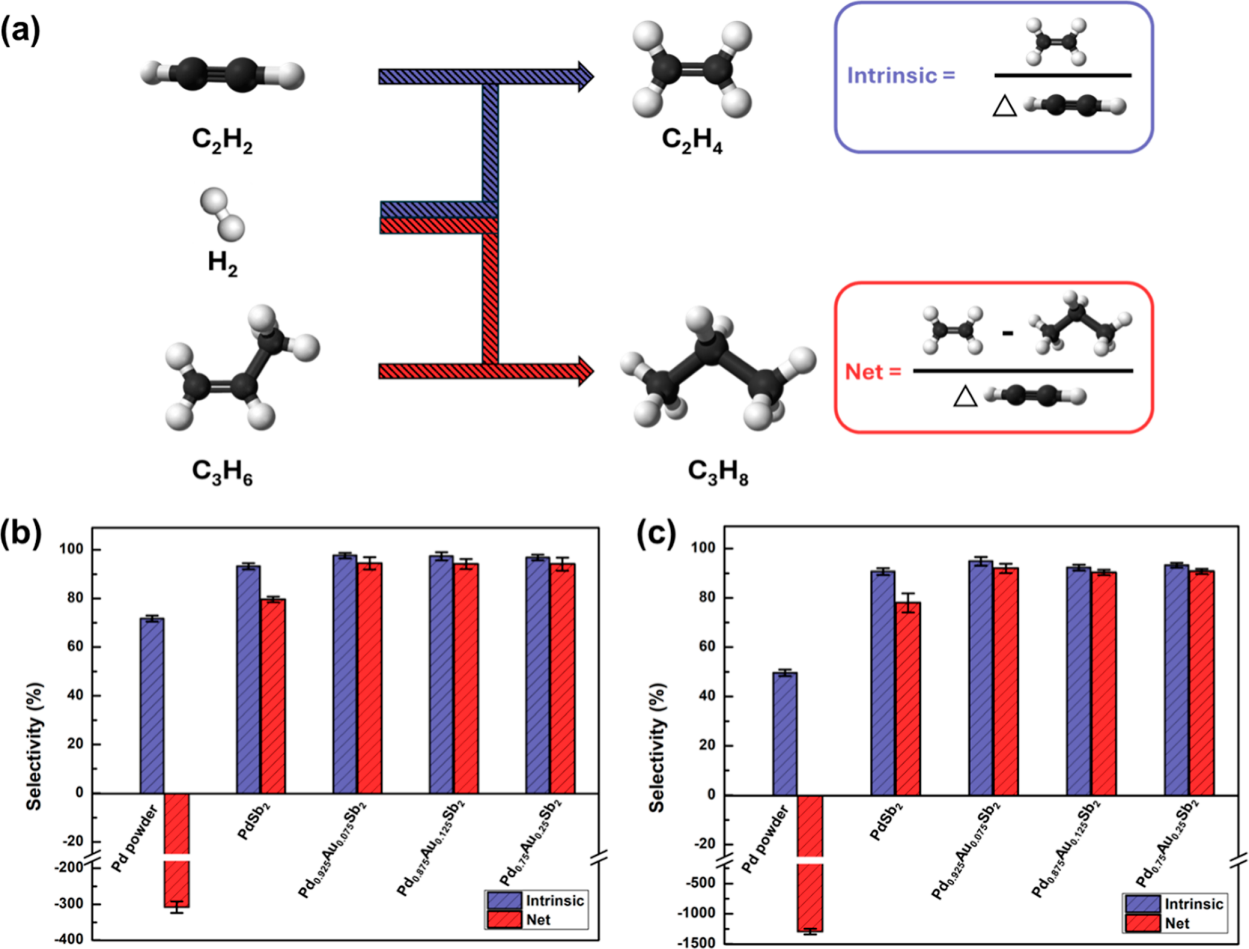
(a) Definition of intrinsic and net selectivity. Intrinsic and net selectivity for Pd powder, PdSb_2_, Pd_0.925_Au_0.075_Sb_2_, Pd_0.875_Au_0.125_Sb_2_ and Pd_0.75_Au_0.25_Sb_2_ at (b) low (20%), and (c) high (90%) acetylene conversion. Acetylene conversion was varied by changes in weight hourly space velocity (WHSV) at a constant reaction temperature of 160 °C. Error bars represent the standard deviation from selectivity measurements using three distinct samples. Reaction conditions are 160 °C and C_3_H_6_/C_2_H_2_/H_2_ = 30:1:200, H_2_: 32 kPa, C_3_H_6_: 4.8 kPa, C_2_H_2_: 0.16 kPa.

Both PdSb_2_ and Pd_1–*x*_Au_*x*_Sb_2_ have similar apparent activation energy barriers for acetylene hydrogenation, with a measured value of ∼ 46 kJ/mol (Figure 4a). The areal acetylene hydrogenation activity of PdSb_2_ is at least doubled (∼2 × 10^–7^ mol m^–2^ s^–1^) compared to Pd_1–*x*_Au_*x*_Sb_2_ (∼1 × 10^–7^ mol m^–2^ s^–1^) at 160 °C (Figure 4b). Given the rate of acetylene hydrogenation does not decrease with increasing Au content suggests the rate of the reaction is dominated by the exposed facet, with the (111) facet exposed on PdSb_2_ demonstrating a higher catalytic activity than the (100) facet (exposed exclusively with Au substitution). Figure S4 demonstrates the propylene and ethylene reaction orders are comparable on PdSb_2_, indicating propylene can act as an appropriate surrogate for ethylene. Both the ethylene and propylene orders over PdSb_2_ were positive (∼0.5), demonstrating weak binding of alkenes on exposed Pd monomers. The propylene order over Pd_1–*x*_Au_*x*_Sb_2_ (Figure 4c) became more positive (∼0.8), which implies even weaker propylene binding, consistent with the superior semihydrogenation selectivity observed for the Au-substituted PdSb_2_ catalysts.

**Figure 4:**
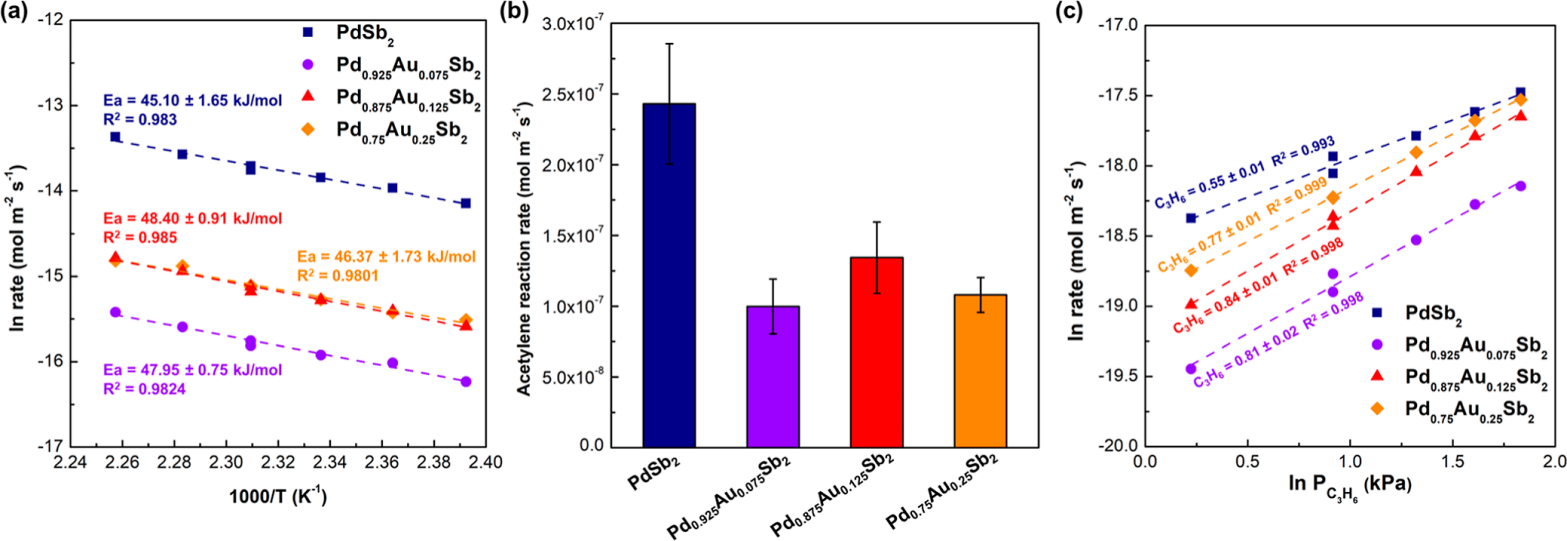
(a) Arrhenius plot of the natural log of acetylene hydrogenation rate over PdSb_2_ (navy square), Pd_0.925_Au_0.075_Sb_2_ (violet circle), Pd_0.875_Au_0.125_Sb_2_ (red triangle) and Pd_0.75_Au_0.25_Sb_2_ (orange rhombus) versus 1000/T. For the apparent activation energy for acetylene hydrogenation, the reaction conditions were 145–170 °C, C_2_H_2_/H_2_ = 1:100, H_2_: 32.5 kPa, and C_2_H_2_: 0.325 kPa. (b) Acetylene reaction rates over PdSb_2_ (navy), Pd_0.925_Au_0.075_Sb_2_ (violet), Pd_0.875_Au_0.125_Sb_2_ (red) and Pd_0.75_Au_0.25_Sb_2_ (orange). Reaction conditions are 160 °C and C_3_H_6_/C_2_H_2_/H_2_ = 30:1:200, H_2_: 32 kPa, C_3_H_6_: 4.8 kPa, C_2_H_2_: 0.16 kPa. Error bars represent the standard deviation from activity measurements using three distinct samples during competitive acetylene-propylene hydrogenation. (c) Plot of the natural log of propylene hydrogenation rate over PdSb_2_ (navy square), Pd_0.925_Au_0.075_Sb_2_ (violet circle), Pd_0.875_Au_0.125_Sb_2_ (red triangle) and Pd_0.75_Au_0.25_Sb_2_ (orange rhombus) versus the natural log of propylene pressure. Propylene orders measured at 160 °C, H_2_: 31.5 kPa and C_3_H_6_ was varied between 1.58 and 7.88 kPa.

We previously discussed the substitution of Pd with Au resulted in the preferential exposure of (100) surfaces and the elimination of (111) surfaces, as confirmed by EBSD and DFT calculations. Elementary reaction energetics were calculated with DFT for acetylene and ethylene hydrogenation on both (100) and (111) surfaces (Figure 5a). The (100) surfaces, with and without Au substitution in the intermetallic, demonstrate a slightly weaker adsorption of acetylene, but a much weaker ethylene adsorption energy, compared to a (111) surface. The activation barrier for the hydrogenation of adsorbed ethylene to adsorbed ethyl is significantly higher than the ethylene desorption energy for the (100) surface, compared to the same energy difference on the (111) surface. This energy difference indicates ethylene is more likely to desorb from the (100) surface rather than undergo further hydrogenation, suggesting the (100) surface will exhibit higher net acetylene hydrogenation selectivity.

**Figure 5:**
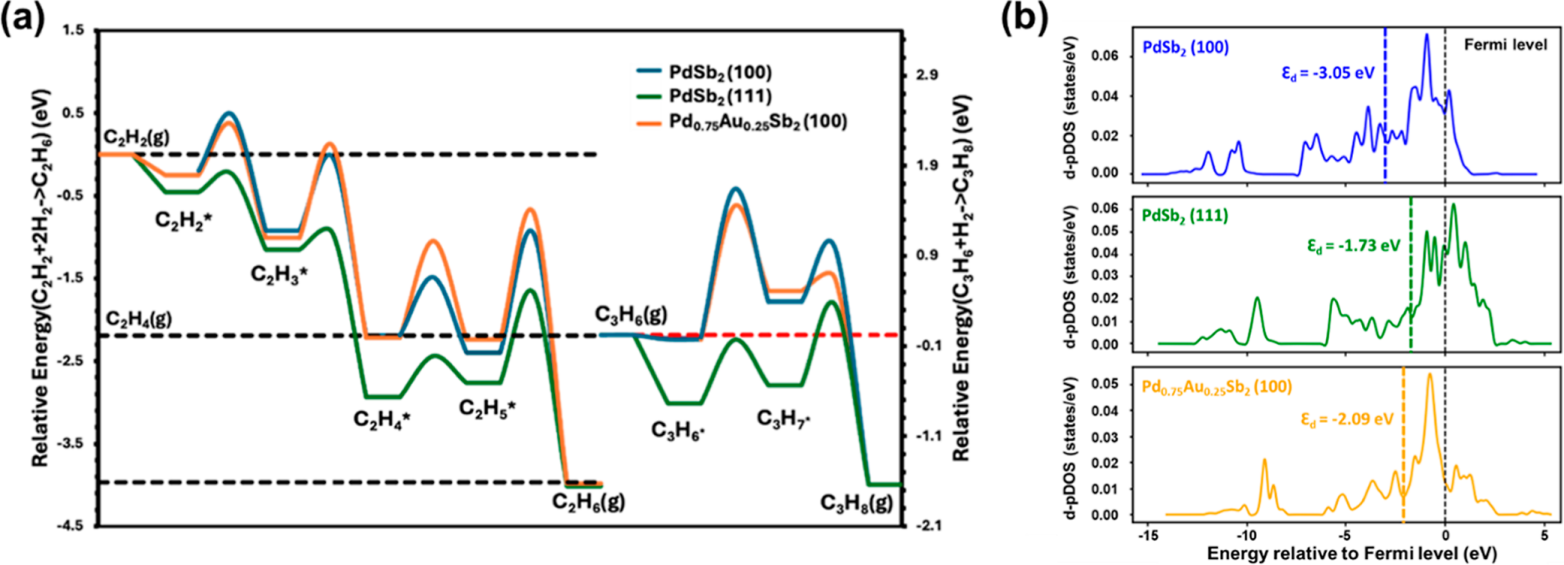
(a) Reaction energetics for competitive acetylene-ethylene and acetylene-propylene hydrogenation over (100) (blue line) and (111) (green line) surfaces of PdSb_2_, and (100) (orange line) surface of Pd_0.75_Au_0.25_Sb_2_. (b) Projected density of states for surface Pd d-states (d-pDOS) of (100) (blue line) and (111) (green line) surfaces of PdSb_2_, and (100) (orange line) surface of Pd_0.75_Au_0.25_Sb_2_.

Surface-energy calculations indicate the lowest-energy (100) termination is Pd-terminated (Figure 2a,b); consequently, the hydrogenation energetics discussed here correspond to Pd surface sites. The (111) surface can expose a termination with partial Au substitution of Pd, and we therefore considered the impact of Au substitution in the surface on the ethylene adsorption energy (Figure S5). The presence of Au on the (111) facet has limited impact on the ethylene binding energy to Pd monomers; Au exposed on the surface will have an insignificant impact on Pd monomer hydrogenation energetics. Comparison of the ethylene selectivity at similar acetylene conversion indicates the improvement in selectivity does not correlate with the number of Pd atoms replaced with Au (a decrease in activity). Rather, the preferential exposure of (100) facet upon Pd substitution dominates the selectivity improvement.

The valence band structure of the surface Pd d-states is consistent with the observed trends in ethylene binding (Figure 5b). The projected Pd d-states at the PdSb_2_ (111) surface (d-band center −1.73 eV below the Fermi level) are centered closer to the Fermi level than for the (100) surface (−3.05 eV), consistent with stronger ethylene binding. The inclusion of Au in the ternary Pd_0.75_Au_0.25_Sb_2_ (100) surface shifts the surface Pd d-states (−2.09 eV) closer to the Fermi level, implying that the alteration in the electronic structure of Pd upon Au substitution would result in stronger ethylene binding and lower net selectivity. Therefore, DFT calculations suggest electronic modification cannot account for the selectivity improvement, indicating the dominant factor is the increased (100) exposure induced by Au substitution of Pd. DFT calculated reaction energies demonstrate the net selectivity is greatly impacted by this facet preference, as the exposure of only (100) surfaces in Pd_1–*x*_Au_*x*_Sb_2_ leads to higher net selectivity, whereas PdSb_2_ shows lower net selectivity owing to the exposure of both (111) and (100) surfaces.

We also performed DFT calculations of the hydrogenation barriers for propylene which provide further evidence that propylene is an appropriate surrogate for ethylene. Consistent with the energetics for ethylene hydrogenation, propylene adsorption is weaker, and hydrogenation is less favored on the (100) surfaces compared to the (111) surfaces (Figure 5a). The reaction energies revealed the first hydrogenation step of propylene resembles that of ethylene and the net selectivity trend remained unchanged between (100) and (111) surfaces, providing reassurance propylene is a suitable surrogate for ethylene, consistent with experimental findings.

Overall, the agreement between experimental and computational data suggests the improvement in the semihydrogenation selectivity is a result of the facet change that occurs upon Pd substitution with Au in Pd_1–*x*_Au_*x*_Sb_2_. This unique result highlights an additional avenue, preferential facet exposure, for tuning the selectivity of intermetallic catalysts for selective hydrogenation reactions.

## 4 Conclusions

We demonstrated the site-specific substitution of Pd with Au in pyrite-type PdSb_2_ results in the formation of stable ternary Pd_1–*x*_Au_*x*_Sb_2_ intermetallics, which preferentially expose the (100) facet over the (111) facet. This facet change upon substitution was confirmed experimentally by EBSD and computationally with DFT calculations. The facet change dictates the semihydrogenation selectivity behavior, which was successfully evaluated in a probe reaction of competitive acetylene-propylene hydrogenation. The (100) facet demonstrated much weaker alkene binding compared to the (111) facet, leading to higher net alkene selectivity. The ability to modulate the exposed facet through chemical substitution provides an additional route to tune the selectivity of bulk intermetallics in selective hydrogenation reactions.

## Supplementary Material



## Notes

Time on stream catalyst behavior; powder X-ray and single crystal X-ray diffraction characterization; elemental analysis by EDS and optical emission spectroscopy; EBSD characterization; alkene reaction orders and DFT calculated adsorbed ethylene structures (PDF)
