# Supplementary material for: Facet Preferencing by Chemical Substitution Controls Semi-Hydrogenation Selectivity in Ternary Pyrite-Type Intermetallic Compounds
Source: ACS Catal. 2026 Jan 28;16(3):2881–90. doi: 10.1021/acscatal.5c08855 (PMC12888016; doi:10.1021/acscatal.5c08855)
Supplement: Supplementary file 1 [file cs5c08855_si_001.pdf]

Supplementary Information

Facet Preferencing by Chemical Substitution Controls Semi-Hydrogenation Selectivity in Ternary Pyrite-type Intermetallics

Mustafa Eid[1], Jin Li[2,‡], Nilanjan Roy[2,‡], Kathryn MacIntosh[2,‡], Michael J. Janik[2,*] and Robert M. Rioux[1,2,*]

[1]*Department of Chemistry, Pennsylvania State University, University Park, PA 16802 (USA)*
[2]*Department of Chemical Engineering, Pennsylvania State University, University Park, PA 16802 (USA)*

*Corresponding author: mjanik@psu.edu, rmr189@psu.edu

‡These authors contributed equally.

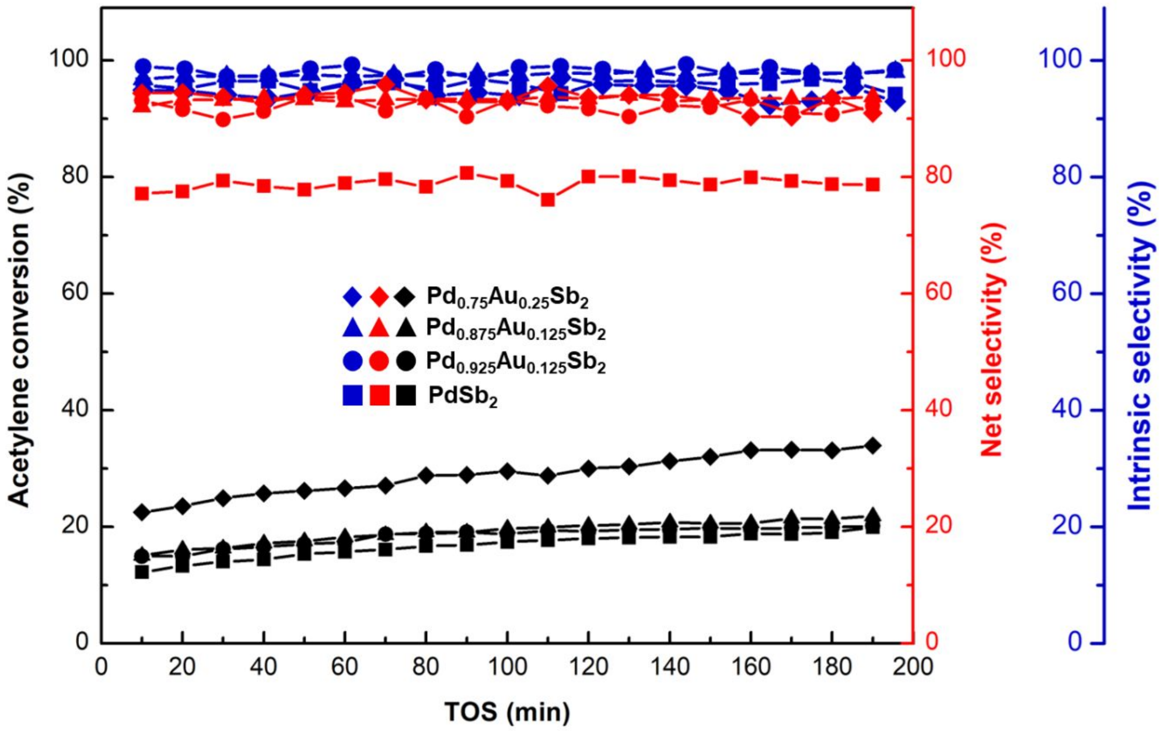

Figure S1. Time on stream behavior of intrinsic selectivity (light navy) and net selectivity (red) at 12 – 34% acetylene conversion (black) during competitive acetylene-propylene hydrogenation over $PdSb_2$ (square), $Pd_{0.925}Au_{0.075}Sb_2$ (circle), $Pd_{0.875}Au_{0.125}Sb_2$ (triangle) and $Pd_{0.75}Au_{0.25}Sb_2$ (rhombus).

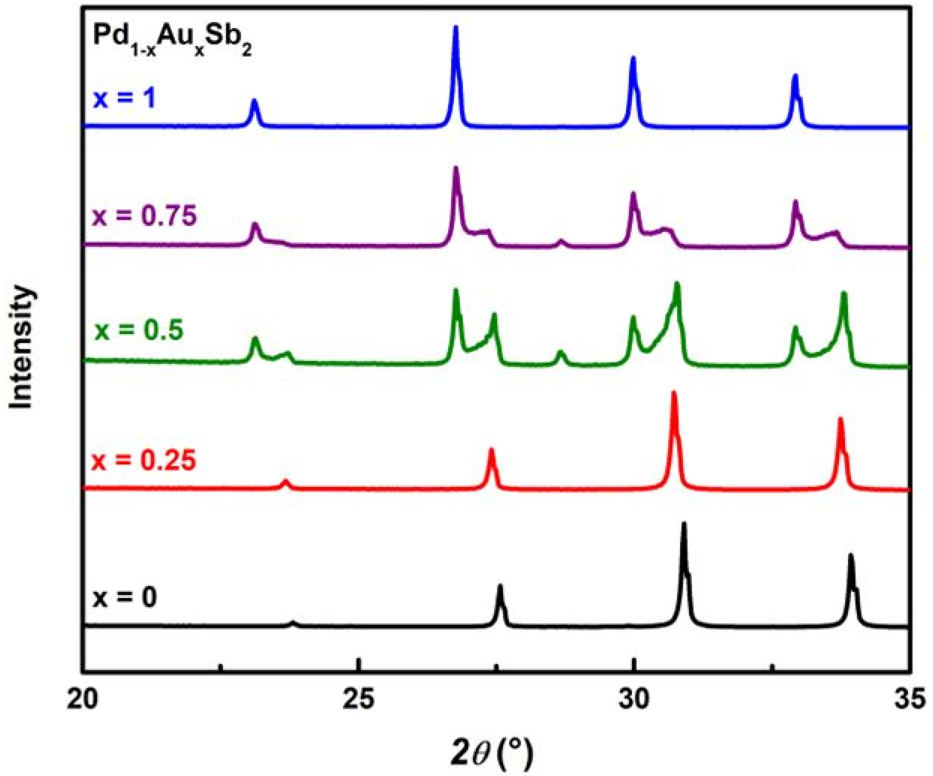

Figure S2. Powder XRD of $Pd_{1-X}Au_xSb_2$ (x = 0, 0.25, 0.5, 0.75 and 1). The entire range of Au substitution (x ≤ 0.25) can be found in Figure 1b of the manuscript.

Table S1. Single crystal refinement for $PdSb_2$, $Pd_{0.925}Au_{0.075}Sb_2$, $Pd_{0.875}Au_{0.125}Sb_2$, $Pd_{0.75}Au_{0.25}Sb_2$, and $AuSb_2$

$PdSb_2$, space group: $Pa\bar{3}$ (205); a = 6.4644 Å, GOF = 1.60, R_{obs} = 2.70					
Atoms	Wyck.	x	y	z	SOF
Pd	4*a*	0	0	0	1
Sb	8*c*	0.3850(1)	0.3850(1)	0.3850(1)	1

$Pd_{0.925}Au_{0.075}Sb_2$, space group: $Pa\bar{3}$ (205); a = 6.4663 Å; GOF = 3.72, R_{obs} = 6.11					
Atoms	Wyck.	x	y	z	SOF
Pd/Au	4*a*	0	0	0	0.927/0.073
Sb	8*c*	0.3850(1)	0.3850(1)	0.3850(1)	1

$Pd_{0.875}Au_{0.125}Sb_2$, space group: $Pa\bar{3}$ (205); a = 6.4668 Å; GOF = 3.11, R_{obs} = 4.54					
Atoms	Wyck.	x	y	z	SOF
Pd/Au	4*a*	0	0	0	0.88/0.12
Sb	8*c*	0.3850(1)	0.3850(1)	0.3850(1)	1

$Pd_{0.75}Au_{0.25}Sb_2$, space group: $Pa\bar{3}$ (205); a = 6.4912 Å; GOF = 2.03, R^2 = 2.65					
Atoms	Wyck.	x	y	z	SOF
Pd/Au	4*a*	0	0	0	0.77/0.23
Sb	8*c*	0.3850(1)	0.3850(1)	0.3850(1)	1

$AuSb_2$, space group: $Pa\bar{3}$ (205); a = 6.6617 Å; GOF = 2.31, R^2 = 3.33					
Atoms	Wyck.	x	y	z	SOF
Au	4*a*	0	0	0	1
Sb	8*c*	0.3850(1)	0.3850(1)	0.3850(1)	1

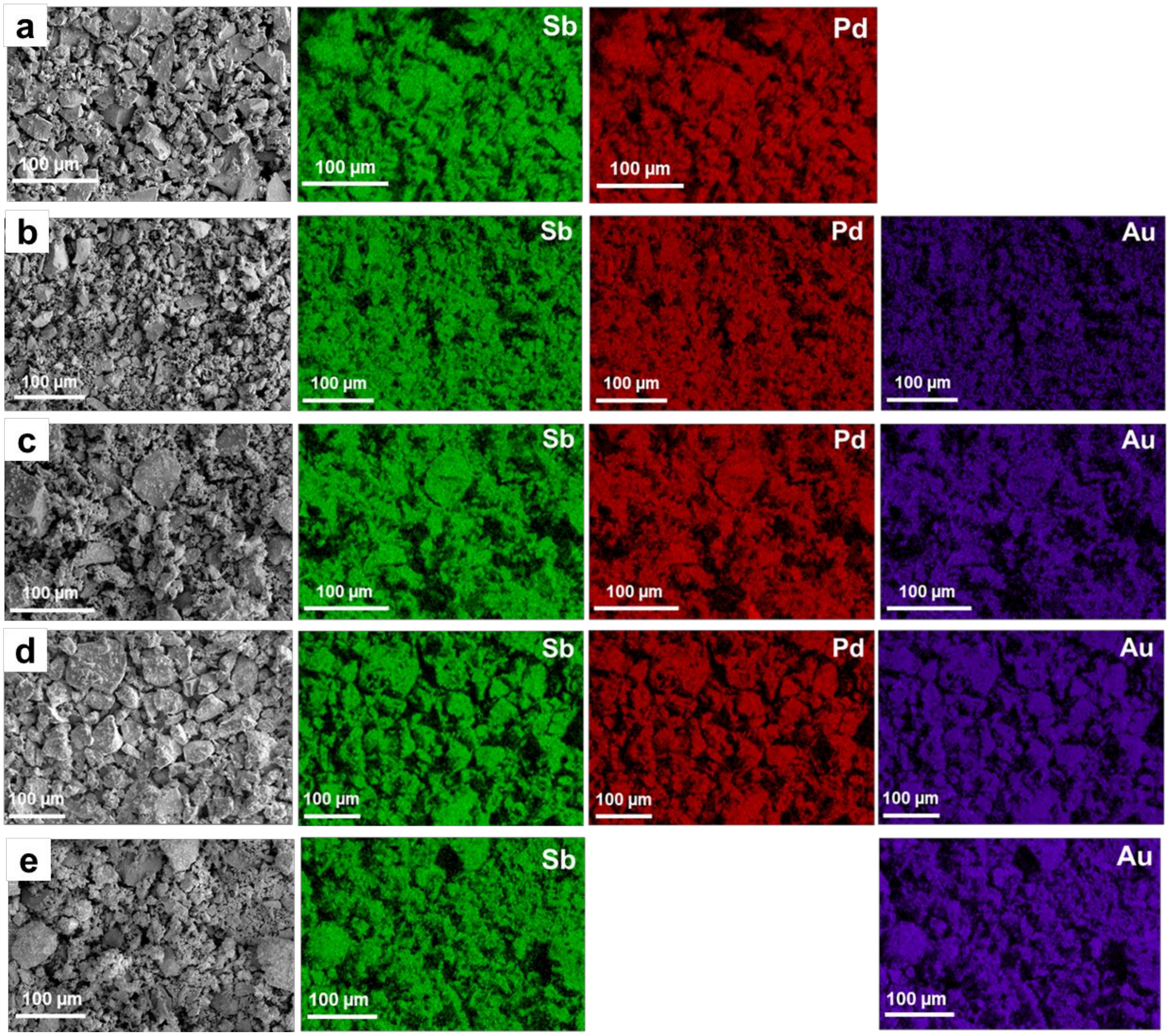

Figure S3. EDS-SEM maps of (a) $PdSb_2$, (b) $Pd_{0.925}Au_{0.075}Sb_2$, (c) $Pd_{0.875}Au_{0.125}Sb_2$, (d) $Pd_{0.75}Au_{0.25}Sb_2$, and (e) $AuSb_2$. Sb (green color), Pd (red color), and Au (deep violet).

Table S2. Metallic compositions for $PdSb_2$, $Pd_{0.925}Au_{0.075}Sb_2$, $Pd_{0.875}Au_{0.125}Sb_2$, $Pd_{0.75}Au_{0.25}Sb_2$, and $AuSb_2$ calculated by EDS-SEM and ICP-OES.

	Elemental analysis					
	ICP			SEM-EDS		
	Pd	Au	Sb	Pd	Au	Sb
$PdSb_2$	0.98	-	2.02	1	-	2
$Pd_{0.925}Au_{0.075}Sb_2$	0.953	0.067	1.98	0.901	0.079	2.02
$Pd_{0.875}Au_{0.125}Sb_2$	0.892	0.118	1.99	0.86	0.13	2.01
$Pd_{0.75}Au_{0.25}Sb_2$	0.77	0.23	2	0.76	0.24	2
$AuSb_2$	-	0.99	2.01	-	1	2

Table S3. Inverse pole figure (IPF) 3D crystal orientation maps for $PdSb_2$, $Pd_{0.925}Au_{0.075}Sb_2$, $Pd_{0.875}Au_{0.125}Sb_2$, and $Pd_{0.75}Au_{0.25}Sb_2$

	X-axis	Y-axis	Z-axis	MUD
$PdSb_2$	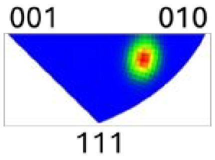	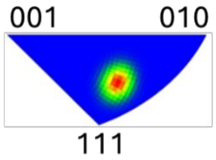	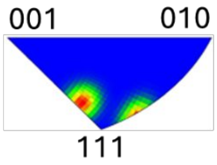	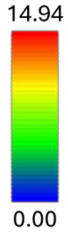
$Pd_{0.925}Au_{0.075}Sb_2$	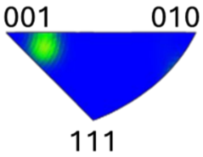	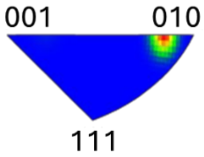	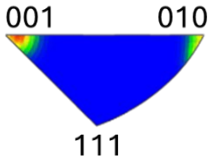	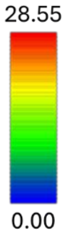
$Pd_{0.875}Au_{0.125}Sb_2$	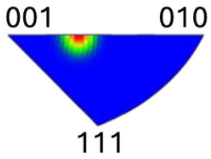	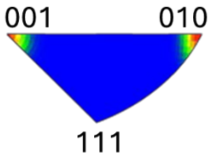	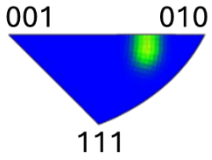	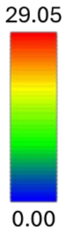
$Pd_{0.75}Au_{0.25}Sb_2$	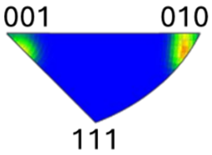	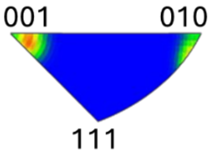	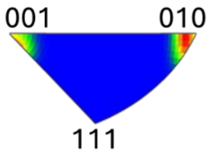	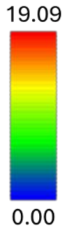

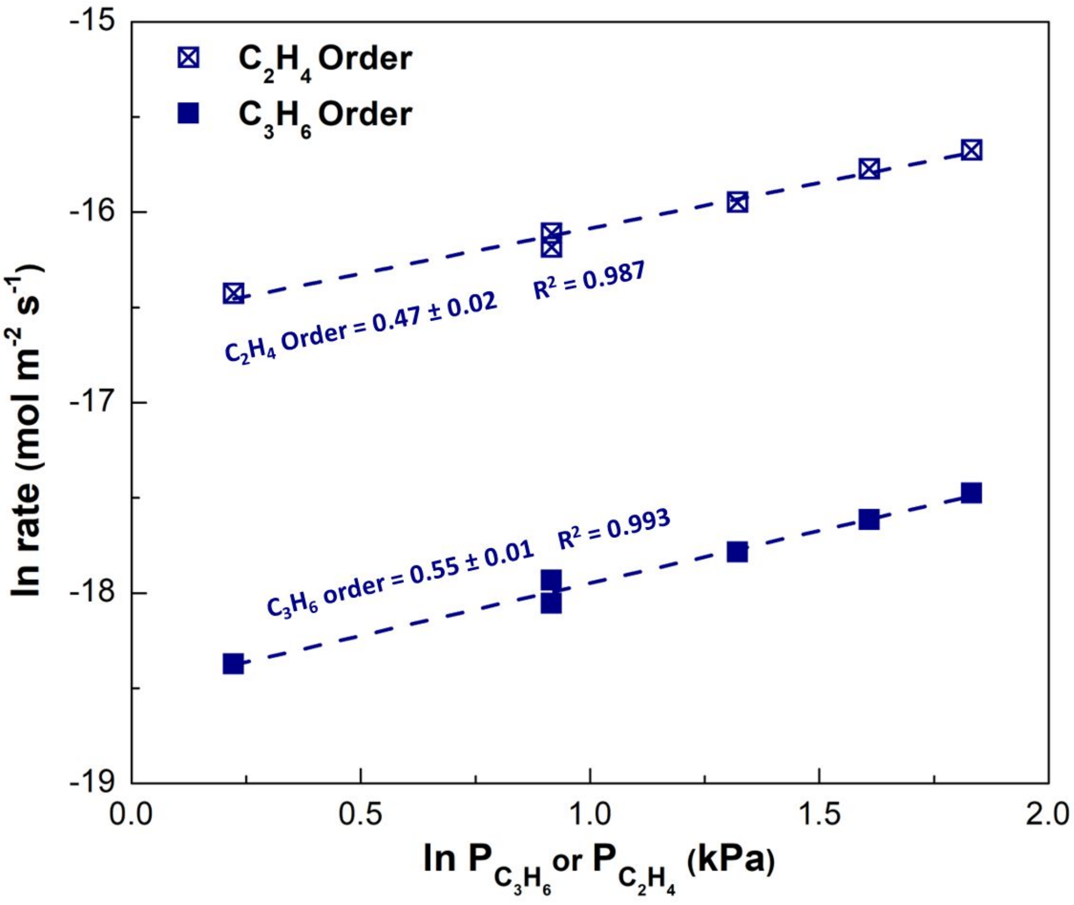

Figure S4. Propylene (filled square) and ethylene orders (cross-marked square) over $PdSb_2$. Propylene order measured at 160 °C, H_2 was held at 31.5 kPa while C_3H_6 varied between 1.58 and 7.88 kPa. Ethylene order measured at 160 °C, H_2 was held at 31.5 kPa while C_3H_6 varied between 1.58 and 7.88 kPa.

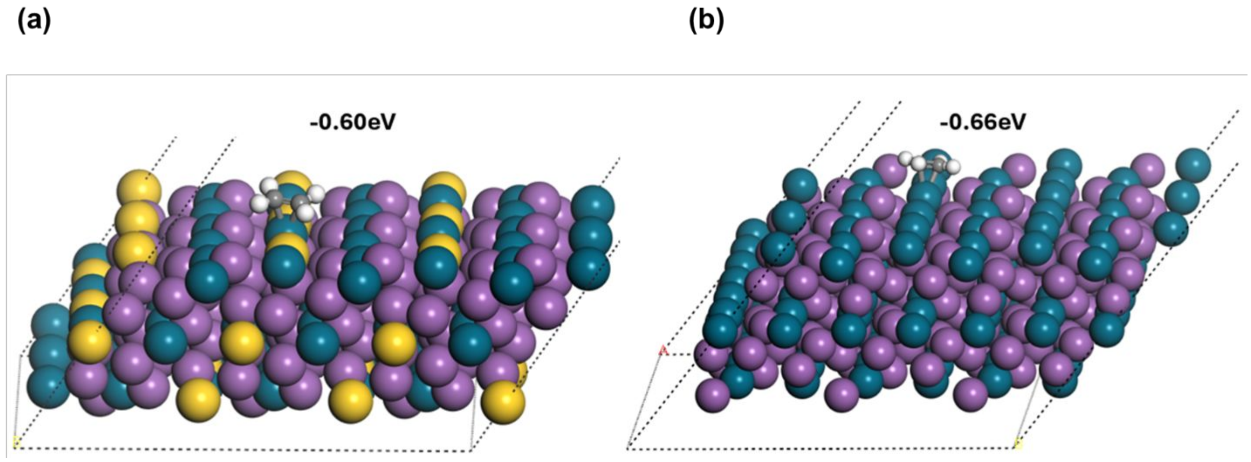

Figure S5. Ethylene adsorption structure and energy on the (111) facet containing (a) Pd and Au sites and (b) Pd sites only. Pd atoms are blue, Sb atoms are violet, and Au atoms are yellow. Ethylene molecule has two carbon atoms (grey) while hydrogen atoms are white.
